# Prognostic values of F-box members in breast cancer: an online database analysis and literature review

**DOI:** 10.1042/BSR20180949

**Published:** 2019-01-03

**Authors:** Xiaochen Wang, Tao Zhang, Shizhen Zhang, Jinlan Shan

**Affiliations:** 1 Department of Surgical Oncology, Second Affiliated Hospital, Zhejiang University School of Medicine, No. 88, Jiefang Road, Hangzhou, Zhejiang 310009, China; 2 Cancer Institute (Key Laboratory of Cancer Prevention & Intervention, National Ministry of Education, Provincial Key Laboratory of Molecular Biology in Medical Sciences), Second Affiliated Hospital, Zhejiang University School of Medicine, No. 88, Jiefang Road, Hangzhou, Zhejiang 310009, China; 3 Institute of Translational Medicine, Zhejiang University School of Medicine, Hangzhou, 310029, Zhejiang, China; 4 Department of oncology, The Affiliated Hospital of Shaoxing University, Shaoxing 312000, Zhejiang, China

**Keywords:** breast cancer, F-box, literature review, prognostic value

## Abstract

**Introduction:** F-box proteins are the substrate-recognizing subunits of SKP1 (S-phase kinase-associated protein 1)–cullin1–F-box protein (SCF) E3 ligase complexes that play pivotal roles in multiple cellular processes, including cell proliferation, apoptosis, angiogenesis, invasion, and metastasis. Dysregulation of F-box proteins may lead to an unbalanced proteolysis of numerous protein substrates, contributing to progression of human malignancies. However, the prognostic values of F-box members, especially at mRNA levels, in breast cancer (BC) are elusive.** Methods:** An online database, which is constructed based on the gene expression data and survival information downloaded from GEO (http://www.ncbi.nlm.nih.gov/geo/), was used to investigate the prognostic values of 15 members of *F-box* mRNA expression in BC. **Results:** We found that higher mRNA expression levels of FBXO1, FBXO31, SKP2, and FBXO5 were significantly associated with worse prognosis for BC patients. While FBXO4 and β-TrCP1 were found to be correlated to better overall survival (OS). **Conclusion:** The associated results provide new insights into F-box members in the development and progression of BC. Further researches to explore the F-box protein-targetting reagents for treating BC are needed.

## Introduction

Ubiquitin proteasome system (UPS) governs diverse cellular processes such as cell proliferation, cell cycle progression, transcription, and apoptosis through targetting specific substrate proteins for ubiquitylation and degradation. The ubiquitin-activating E1 enzyme, ubiquitin–conjugating E2 enzyme and ubiquitin-protein E3 ligase exert the multistep enzymatic processes to catalyze the ubiquitinated substrates. The SKP1–cullin1–F-box protein (SCF) E3 ligase complex, which is composed of the invariant components S-phase kinase-associated protein 1 (SKP1), the E3 ligase RBX1 (also known as ROC1) and cullin 1, as well as variable F-box proteins [1], is so far the best characterized E3 ligase family member [2]. The F-box proteins are able to bind to a distinct subset of substrates though its WD40 or leucine-rich domains and determine the substrate specificity of SCF complex [3]. Until now, 69 mammalian F-box proteins have been identified, they can be organized into three subclasses [4]: (i) the well-studied β-TRCP1, FBXW7 (also known as Fbw7, Sel-10, hCdc4, or hAgo), and β-TRCP2 (also known as FBXW11), which contain WD40 repeat domains; (ii) FBXL family members, including SKP2 (also known as FBXL1), which contain leucine-rich repeat domains; and (iii) FBXO proteins. Owing to the pivotal and indispensable roles in cell cycle regulation that have been identified, the relationship between these proteins and tumorigenesis attract much attention [5].

Breast cancer (BC) is the most common malignant disease that causes the most cancer-related deaths amongst females worldwide [6]. According to the expression patterns of hormone and growth factor receptors, BCs are classified into four major molecular subtypes: luminal A and B, HER2-like, and basal-like. Due to the heterogeneous and high morbidity of disease, the death rates of BC remain high [7]. Therefore, the detailed molecular mechanism underlying the BC development and progression is important to be explored, and it is essential to identify novel targets for predicting or treating BCs. Amongst the 69 F-box proteins, only four members—FBXW7, SKP2, β-TrCP1, and β-TrCP2—have been extensively studied, and 15 of them are so far identified to play determined roles in cancers and they are grouped into four categories: tumor suppressive, oncogenic, context-dependent, or undetermined functions in cancer [4]. Nevertheless, the prognostic values of each individual F-box proteins, specially at the mRNA level in BCs are still elusive.

Kaplan–Meier plotter (KM plotter) database is constructed based on the gene expression data and survival information downloaded from GEO (http://www.ncbi.nlm.nih.gov/geo/) [8]. Owing to its ease of access to database, this online survival analysis tool has been widely used to analyze the prognostic values of individual genes in lung cancer, ovarian cancer, gastric cancer, and BC [9–12]. In the present study, we selected 15 well-identified members of F-box family to assess their prognostic values for BC. The relationship between F-box mRNA expression and clinical characteristics were also analyzed by KM plotter database.

## Materials and methods

An online KM plotter database [8] was used to assess the prognostic values of 15 F-box members’ mRNA expression in BC as previously described [11]. The background database of this online survival analysis tool was established using gene expression data and survival information of 1809 patients (1402 BC patients with overall survival (OS) data) downloaded from GEO (Affymetrix HGU133A and HGU133+2 microarrays) [8]. These two microarrays are frequently used because these two arrays contain 22277 probe sets at nearly identical platforms. An overview of the clinical data is presented in Table 1 [13–25]. Each of 15 individual members of F-box members were entered into this online analysis database respectively (http://kmplot.com/analysis/index.php?p=service&cancer=breast), and Kaplan–Meier survival curves were acquired. Hazard ratio (HR), 95% confidence intervals (CI), and log rank *P*-values were also obtained on the webpages, and *P-*values of <0.05 were considered as statistically significant.

**Table 1:** Clinical characteristics of the microarray datasets used in the analysis

GEO ID	Platform	Number of patients	Age (years)	Tumor size (cm)	ER+	Lymph node+	Grade 1	Grade 2	Grade 3	Relapse event	Average relapase-free survival	References
GSE12276	GPL570	204	NA	NA	NA	NA	NA	NA	NA	204	2.2 ± 1.8	Bos et al. (2009)
GSE16391	GPL570	55	61 ± 9	NA	55	33	2	35	18	55	3.0 ± 1.2	Desmedt et al. (2009)
GSE12093	GPL96	136	NA	NA	136	0	NA	NA	NA	20	7.7 ± 3.2	Zhang et al. (2009)
GSE11121	GPL96	200	NA	2.1 ± 1	NA	0	58	136	35	46	7.8 ± 4.2	Schmidt et al. (2008)
GSE9195	GPL570	77	64 ± 9	2.4 ± 1	77	36	14	20	24	13	7.8 ± 2.5	Loi et al. (2008)
GSE7390	GPL96	198	46 ± 7	2.2 ± 0.8	134	NA	30	83	83	91	9.3 ± 5.6	Desmedt et al. (2007)
GSE6532	GPL96	82	64 ± 10	2.5 ± 1.2	70	22	0	54	1	19	6.1 ± 3.1	Loi et al. (2007)
GSE5327	GPL96	58	NA	NA	0	NA	NA	NA	NA	11	6.8 ± 3.1	Minn et al. (2007)
GSE4922	GPL96	1	69	2.2	1	0	1	0	0	0	12.17	Ivshina et al. (2006)
GSE2494	GPL96	251	62 ± 14	2.2 ± 1.3	213	84	67	128	54	NA	NA	Miller et al. (2005)
GSE2990	GPL96	102	58 ± 12	2.3 ± 1.1	73	15	27	20	36	40	6.6 ± 3.9	Sotirious et al. (2006)
GSE2034	GPL96	286	NA	NA	209	0	NA	NA	NA	107	6.5 ± 3.5	Wang et al. (2005)
GSE1456	GPL96	159	NA	NA	NA	NA	28	58	61	40	6.2 ± 2.3	Pawitan et al. (2005)
Total		1809	57 ± 13	2.2 ± 1.1	968	190	227	534	312	646	6.4 ± 4.1	

## Results

### Prognostic roles of F-box in all BC patients

**Figure 1:**
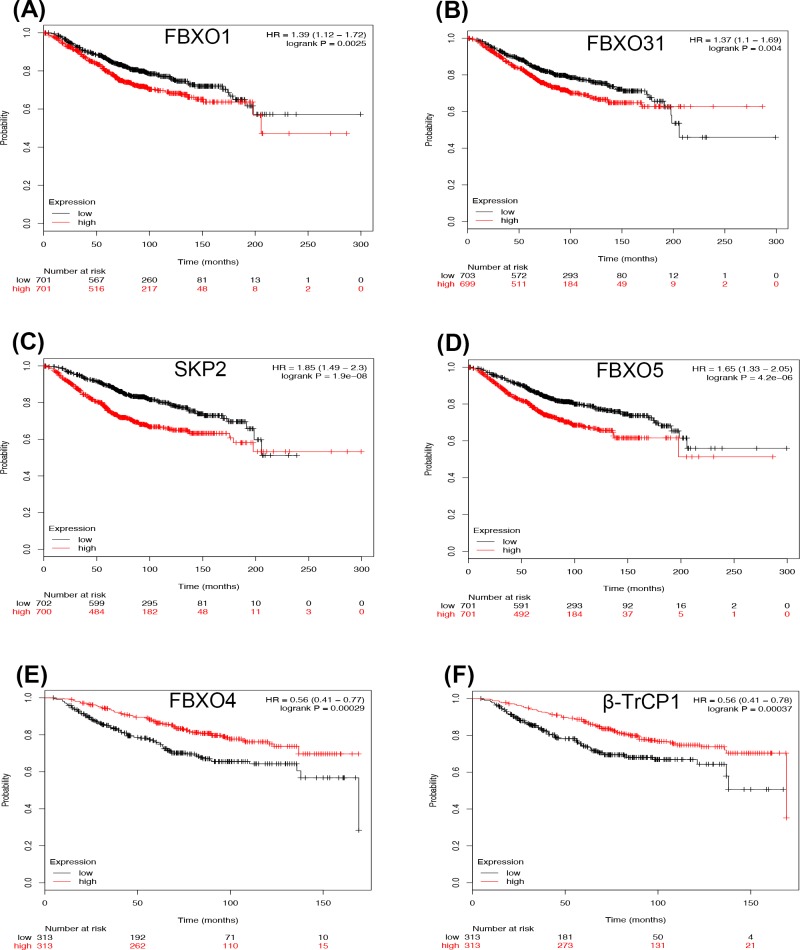
The prognostic values of the mRNA expression of F-box in all BCs Overexpression of FBXO1 (**A**), FBXO31 (**B**), SKP2 (**C**), and FBXO5 (**D**) are significantly associated with worse OS in all BC patients. Overexpression of FBXO4 (**E**) and β-TrCP1 (**F**) are associated with better prognosis.

We first examined the prognostic effects of 15 members of *F-box* mRNA in all BC patients by KM plotter database. As shown in Figure 1, FBXO1 (HR = 1.39 95%CI: 1.12–1.72, *P*=0.0025), FBXO31 (HR = 1.37 95%CI: 1.10–1.69, *P*=0.0040), SKP2 (HR = 1.85 95%CI: 1.49–2.30, *P*=0.0008), and FBXO5 (HR = 1.65 95%CI: 1.33–2.05, *P*=0.0004) were significantly associated with worse OS in all BC patients (Figure 1A–D). However, FBXO4 (HR = 0.56 95%CI: 0.41–0.77, *P*=0.0003) and β-TrCP1 (HR = 0.73 95%CI: 0.59–0.90, *P*=0.0034) were associated with better prognosis (Figure 1E,F). The mRNA expression levels of FBXW8, FBXL3, FBXO10, FBXO11, FBXO18, FBXO9, β-TrCP2, and FBXL10 were not correlated with OS in all BC patients (Supplementary Figure S1).

### Prognostic roles of F-box members in different BC subtypes

**Figure 2:**
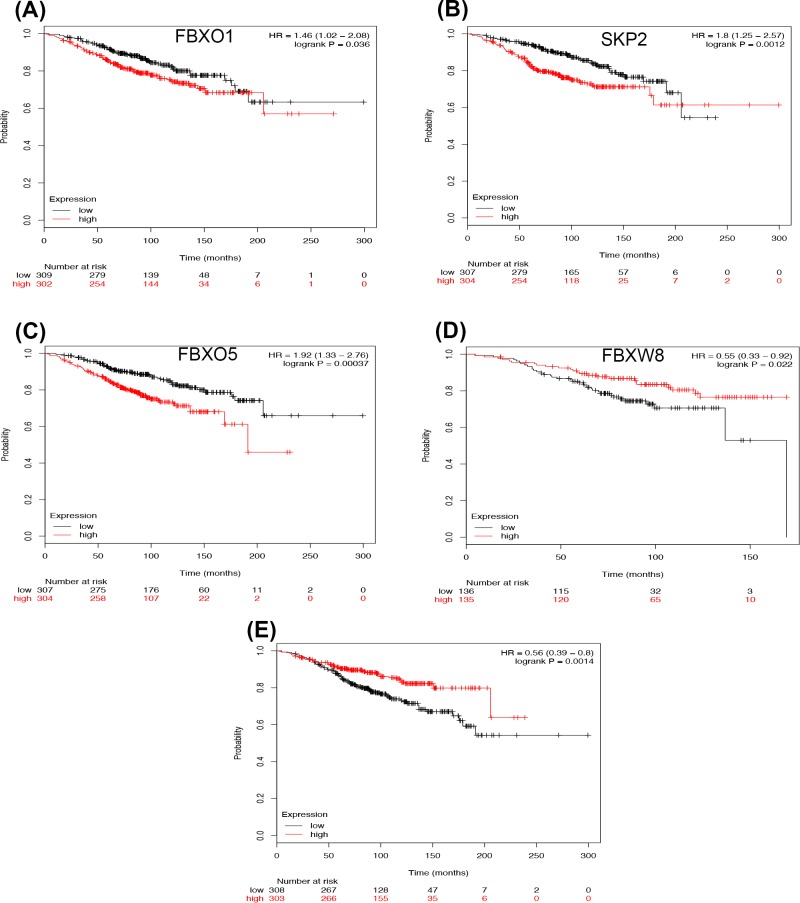
The prognostic values of the mRNA expression of F-box in luminal A type BCs The high expression of FBXO1 (**A**), SKP2 (**B**), and FBXO5 (**C**) are correlated to worse survival, and FBXW8 (**D**) and β-TrCP1 (**E**) are associated with longer OS in luminal A type BC patients.

Then, we respectively assessed the prognostic effects of F-box in BCs with different intrinsic subtypes. For luminal A type BC patients, FBXO1 (HR = 1.46 95%CI: 1.02–2.08, *P*=0.0358), SKP2 (HR = 1.80 95%CI: 1.25-2.57, *P*=0.0012), and FBXO5 (HR = 1.92 95%CI: 1.33–2.76, *P*=0.0004) were correlated to worse survival (Figure 2A–C). Whereas FBXW8 (HR = 0.55 95%CI: 0.33–0.92, *P*=0.0219) and β-TrCP1 (HR = 0.56 95%CI: 0.39–0.80, *P*=0.0014) were significantly associated with longer OS (Figure 2D,E). The rest members of F-box were not correlated to prognosis in luminal A type BC (Supplementary Figure S2).

**Figure 3:**
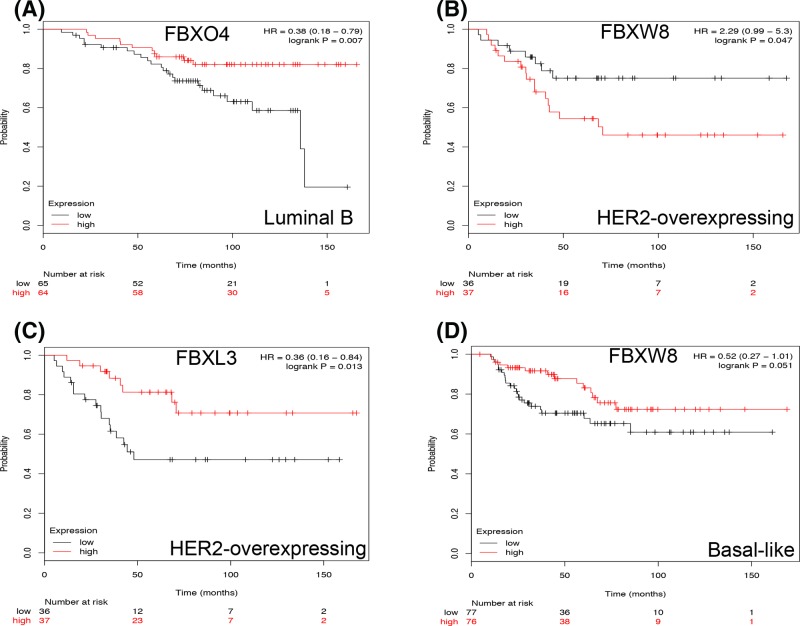
The prognostic values of some selected F-box in luminal B type, HER2-expressing or Basal-like BCs Survival curves of FBXO4 (**A**) are plotted for luminal B type BC patients. Survival curves of FBXW8 (**B**) and FBXL3 (**C**) are plotted for HER2-overexpressing BC patients. Survival curves of FBXW8 (**D**) are plotted for basal-like BC patients.

In luminal B type BC patients, only high mRNA expression of FBXO4 was significantly associated with better survival, the HR was 0.38 (95%CI: 0.18-0.79, *P*=0.0070, Figure 3A). The remaining F-box members did not show any prognostic value in luminal B type BC patients (Supplementary Figure S3).

In HER2-overexpressing BC, high mRNA expression of FBXW8 was associated with poor prognosis, the HR was 2.29 (95%CI: 0.99, 5.30), *P*=0.0475 (Figure 3B). However, FBXL3 (HR = 0.36, 95%CI: 0.16–0.84, *P*=0.0134) was significantly associated with better OS (Figure 3C). The rest of F-box members were not associated with prognosis in HER2-overexpressing BC patients (Supplementary Figure S4).

With regard to basal-like BC, none of the selected F-box members was associated with prognosis (Supplementary Figure S5). Only FBXW8 (HR = 0.52, 95%CI: 0.27–1.01, *P*=0.051) was modestly associated with better prognosis (Figure 3D).

### Prognostic roles of F-box members in BC patients with different status of TP53

Furthermore, we assessed prognostic values of F-box members in BCs with different status of TP53. As shown in Table 2, only SKP2 (HR = 1.79, 95%CI: 0.92–3.49, *P*=0.0809) was modestly associated with worse survival for wild-TP53-type BCs, the other F-box members were not correlated with prognosis. In mutant-TP53-type BC, FBXL3 was significantly associated with longer OS, however, the other F-box members did not show any prognostic values.

**Table 2:** The association between the F-box members and the prognosis of BC with different p53 status

F-box family	Affymetrix IDs	p53	HR	95%CI	*P*-value
FBXW7	229419_at	Mutant	1.99	(0.50, 7.98)	0.3193
		Wild	/	/	/
FBXO4	223493_at	Mutant	1.20	(0.32, 4.48)	0.7888
		Wild	/	/	1.88)/
FBXW8	231883_at	Mutant	1.01	(0.26, 3.90)	0.9886
		Wild	/	/	/
FBXL3	225132_at	Mutant	0.11	(0.01, 0.91)	0.0136
		Wild	1.28	(0.55, 3.01)	0.5648
FBXO1	204826_at	Mutant	0.94	(0.44, 2.01)	0.8786
		Wild	0.87	(0.46, 1.66)	0.6758
FBXO10	227222_at	Mutant	0.83	(0.22, 3.07)	0.7745
		Wild	/	/	/
FBXO11	222119_s_at	Mutant	0.83	(0.38, 1.82)	0.6423
		Wild	0.57	(0.29, 1.12)	0.1004
FBXO18	224683_at	Mutant	0.50	(0.13, 2.02)	0.3248
		Wild	/	/	/
FBXO31	219785_s_at	Mutant	0.53	(0.24, 1.19)	0.1201
		Wild	0.98	(0.51, 1.86)	0.9411
SKP2	203625_at	Mutant	0.70	(0.33, 1.52)	0.3681
		Wild	1.79	(0.92, 3.49)	0.0809
FBXO5	218875_at	Mutant	1.02	(0.46, 2.27)	0.9562
		Wild	1.24	(0.92, 3.49)	0.0809
FBXO9	238472_at	Mutant	1.93	(0.48, 7.73)	0.3471
		Wild	/	/	/
β-TrCP1	216091_s_at	Mutant	1.47	(0.63, 3.43)	0.3684
		Wild	1.01	(0.53, 1.92)	0.9799
β-TrCP2	209455_at	Mutant	0.79	(0.37, 1.71)	0.5514
		Wild	1.52	(0.79, 2.92)	0.2037
FBXL10	226215_s_at	Mutant	1.43	(0.38, 5.41)	0.6004
		Wild	/	/	/

### Prognostic roles of F-box members in BC patients with different pathological grades

Next, we assessed prognostic values of F-box members in different pathological grade BCs. We could see from the Table 3 that none of the F-box members was found to be associated with prognosis in grade I BC patients. While in grade II BC, FBXW7 (HR = 0.27, 95%CI: 0.07–1.02, *P*=0.0383) was correlated with better OS, FBXO1 (HR = 2.10, 95%CI: 1.34–3.30, *P*=0.0001) and SKP2 (HR = 1.56, 95%CI: 1.01–2.40, *P*=0.0420) were significantly associated with poor survival. However, the higher mRNA expression of FBXO4 (HR = 0.59, 95%CI: 0.35-0.99, *P*=0.0430) and FXBL3 (HR = 0.52, 95%CI: 0.31–0.88, *P*=0.0136) were associated with better survival for grade III BCs.

**Table 3:** Correlation of F-box with different pathological grade status of BC patients

F-box family	Affymetrix IDs	Grades	HR	95%CI	*P-*value
FBXW7	229419_at	I	0.57	(0.05, 6.27)	0.6390
		II	0.27	(0.07, 1.02)	0.0383*
		III	1.32	(0.79, 2.21)	0.2833
FBXO4	223493_at	I	1.66	(0.15, 18.35)	0.6780
		II	1.10	(0.35, 3.48)	0.8711
		III	0.59	(0.35, 0.99)	0.0430*
FBXW8	231883_at	I	0.26	(0.02, 3.57)	0.2910
		II	0.94	(0.30, 2.92)	0.9179
		III	0.79	(0.48, 1.32)	0.3707
FBXL3	225132_at	I	2.02	(0.18, 22.59)	0.5610
		II	0.80	(0.25, 2.51)	0.6973
		III	0.52	(0.31, 0.88)	0.0136*
FBXO1	225132_at	I	0.73	(0.28, 1.87)	0.5070
		II	2.10	(1.34, 3.30)	0.0001*
		III	0.91	(0.66, 1.27)	0.5884
FBXO10	227222_at	I	0.44	(0.04, 4.86)	0.4900
		II	0.87	(0.28, 2.70)	0.8039
		III	1.58	(0.95, 2.64)	0.0768
FBXO11	222119_s_at	I	0.66	(0.26, 1.67)	0.3750
		II	0.89	(0.58, 1.38)	0.6109
		III	1.37	(0.98, 1.91)	0.0628
FBXO18	224683_at	I	1.76	(0.16, 19.53)	0.6390
		II	0.59	(0.18, 1.97)	0.3872
		III	0.81	(0.49, 1.36)	0.4288
FBXO31	219785_s_at	I	1.11	(0.45, 2.76)	0.8187
		II	1.22	(0.79, 1.87)	0.3661
		III	1.32	(0.95, 1.83)	0.0953
SKP2	203625_at	I	1.59	(0.65, 3.92)	0.3078
		II	1.56	(1.01, 2.40)	0.0420*
		III	1.01	(0.73, 1.40)	0.9648
FBXO5	218875_s_at	I	1.65	(0.67, 4.07)	0.2727
		II	1.41	(0.92, 2.17)	0.1110
		III	1.38	(0.99, 1.91)	0.0570
FBXO9	238472_at	I	0.57	(0.05, 6.27)	0.6390
		II	1.91	(0.58, 6.37)	0.2812
		III	0.98	(0.58, 1.65)	0.9426
β-TrCP1	216091_s_at	I	0.60	(0.24, 1.51)	0.2719
		II	1.01	(0.66, 1.55)	0.9678
		III	0.84	(0.60, 1.16)	0.2890
β-TrCP2	209455_at	I	0.88	(0.35, 2.20)	0.7763
		II	1.04	(0.68, 1.59)	0.8656
		III	1.30	(0.94, 1.81)	0.1155
FBXL10	226215_s_at	I	0.50	(0.04, 5.54)	0.5610
		II	0.51	(0.15, 1.70)	0.2661
		III	0.66	(0.39, 1.11)	0.1146

## Discussion

F-box protein is one of the core components of SCF multisubunit E3 ligase complex, it determines the substrate specificity of SCF complex by binding to substrates through WD40 or leucine-rich domains [3]. F-box family members are divided into three subclasses, including 10 FBXW proteins, 22 FBXL proteins, and 37 FBXO proteins. F-box proteins are implicated in multiple cellular processes, including cell proliferation, apoptosis, angiogenesis, and invasion via mediating degradation of numerous substrates [4]. In this study, by using an online survival analysis tool, we found that high mRNA expression of FBXO4 and β-TrCP1 were associated with better outcome for BCs, and FBXO1, FBXO31, FBXO5, and SKP2 were significantly correlated to worse prognosis.

FBXO4 is generally identified as a tumor suppressor, FBXO4-deficient mice will develop highly aggressive melanomas, as well as lymphomas, histolytic sarcomas, mammary and hepatocellular carcinomas [26,27]. Mutation or loss of FBXO4 impairs the dimerization of the SCF^Fbx4^ ligase, resulting in accumulation of nuclear cyclin D1 and oncogenic transformation [28–30]. However, how FBXO4 determinates the cell fates of BC cells is unclear. We searched the Pubmed database and did not find any articles on the relationship between FBXO4 and BC. Hence, we used the KM plotter database to analyze the prognostic effect of FBXO4 in BC and found that high mRNA expression of FBXO4 was associated with longer OS for all BC patients. Additionally, high *FBXO4* mRNA expression was correlated to better survival in luminal B and grade III BC patients.

β-TRCP1 and β-TRCP2 either exert their oncogenic or tumor suppressive roles depending on the specific cellular context(s). Interestingly, female mice with β-TRCP1^−/−^ mammary glands exhibited hypoplastic phenotypes, which suggested that β-TRCP1 was critical for tissue development [31]. β-TRCP1 was significantly up-regulated in prostate cancer and hepatoblastoma [32], and high expression of β-TRCP1 at both mRNA and protein levels in colorectal cancer were correlated with poor clinical prognosis [33]. However, in gastric cancers, somatic mutation of β-TRCP1, which impaired ligase activity, contributed to tumor development and progression through β-catenin stabilization [34]. In TNBC cells, knockdown of β-TRCP1 reduced the cell proliferative ability [35], implicating a tumor suppressive role of β-TRCP1 in BC. Here, we showed that high mRNA expression of β-TRCP1 was associated with longer OS in luminal A type BC or all BC patients. On the other hand, β-TRCP2 also has tumor type-dependent roles in dominating tumorigenesis. Overexpression of β-TRCP2 was observed in a variety of human cancers, including prostate, breast, and gastric cancers [36]. Whereas mutation of β-TRCP2 in gastric cancer caused β-catenin accumulation, and contributed to carcinogenesis by activating WNT signaling pathway [37]. Inhibition of β-TRCP2 by miR-106b-25 cluster in non-small lung cancer cells promoted cell invasion and metastasis [38]. However, β-TRCP2 was not associated with prognosis in BC patients according to the current results analyzed by KM plotter database.

FBXO1, also known as cyclin F, meditates centrosome duplication and is critical for maintaining genome integrity, thus it has been regarded as an emerging tumor suppresser [39]. Knockout FBXO1 in MEFs leads to cell cycle defects [40]. In hepatocellular carcinoma, FBXO1 was down-regulated and low expression levels of FBXO1 were significantly associated with worse clinical characteristics and poorer prognosis [41]. Unexpectedly, we suggested an uncanonical function of FBXO1 exerted in BC, as we showed that high mRNA expression levels of FBXO1 were associated with worse survival in BC patients.

FBXO31 was regarded as an emerging tumor suppressor, which is often down-regulated in several human cancers, including BC, gastric cancer, and hepatocellular cancer [42–44]. FBXO31 was involved in DNA damage response for maintaining genomic stability. After DNA damage induced by genotoxic agents or γ-irradiation, phosphorylation of FBXO31 was increased immediately [45], then SCF/FBXO31 promoted MDM2 ubiquitination, resulting in accumulation of p53 and growth arrest [46]. Overexpression of FBXO31 in cancer cells inhibited cell growth and colon formation, and ectopic expression of FBXO31 significantly decreased tumor formation in xenograft nude mice [42–44]. However, overexpression of FBXO31 in lung cancer promoted cell growth and metastasis [47], and higher expression levels of FBXO31 predicted worse survival in esophageal squamous cell carcinoma [48]. Therefore, FBXO31 may also exert its role in tumorigenesis depending on tumor cell types. In our study, we used the KM plotter database to reveal that higher expression of *FBXO31* mRNA was associated with poorer prognosis in BC patients.

The F-box protein SKP2 plays an oncogenic role in human cancers. Mechanistically, SKP2 facilitates ubiquitination and degradation of many tumor suppressors, such as p21, p27, p57, FOXO1, and others [2]. Furthermore, SKP2 enhances DNA damage response and promotes DNA double-strand break repair pathways in cancer cells [49]. As a result, SKP2 is up-regulated in several human cancers, including colorectal cancer [50], bladder cancer [51], BC [52,53], melanoma [54], prostate cancer [55], hepatocellular cancer [56], cervical cancer [49], and lymphoma [57]. In BC, SKP2 has been reported to correlate to poorer prognosis [58–60]. Additionally, immunohistochemical analysis indicated that overexpression of SKP2 were more frequently observed in ER-negative BC [53,59]. In our study, the higher expression of *SKP2* mRNA was significantly associated with shorter OS in luminal A type BC patients. Previous results have indicated that SKP2 expression was associated with higher tumor grade in BCs or bladder cancers [51,59,60]. Interestingly, we showed that high mRNA expression of SKP2 was correlated to poor prognosis in grade II BC patients, but not in grade I or grade III BC patients.

FBXO5 is also suggested to play an emerging oncogenic role in human cancers. FBXO5 functions as an endogenous inhibitor of APC/C, which results in the stabilization of APC/C ubiquitin substrates, such as cyclin A, cylcin B, or Secure [61]. Up-regulation of FBXO5 in p53-deficient cells could promote cell proliferation, tetraploidy, and genomic instability [62]. By analysis of more than 1600 benign and malignant tumors, Lehman et al. [61] suggested that FBXO5 was strongly overexpressed in malignant tumors, rather than in benign tumors. Furthermore, overexpression of FBXO5 was associated with poor outcome in ovarian cancer [63], prostate cancer [64], and hepatocellular carcinoma [65]. In BC patients, overexpression of FBXO5 was significantly correlated with histologic grade and prognosis [66]. Consistently, our results demonstrated that FBXO5 had an oncogenic role in BC, higher expression of FBXO5 in mRNA level was significantly associated with poorer survival, especially in luminal A type BC patients.

FBXO11 was able to inhibit tumor cell growth and induced cell death by target BCL-6 for degradation [67], and deletion or mutation of FBXO11 in pancreatic cancer patients was associated with poor prognosis [68]. In BCs, FBXO11 restrained tumor initiation and metastasis by promoting SNAIL ubiquitylation and degradation, and overexpression of FBXO11 was correlated with longer metastasis-free survival [69,70]. However, our results did not find any relationship between the *FBXO11* mRNA expression and OS in BC.

FBXW8 forms a functional E3 ligase complex with cullin 7 to exert a tumor suppressive role [4]. Ectopic expression of FBXW8 in choriocarcinoma JEG-3 cells increased the percentage of cells at S-phase and decreased the percentage of G_2_/M-phase cells [71], suggesting that FBXW8 was critical for cell growth. As FBXW8-meditated cyclin D1 and HPK1 degradation was necessary for cancer cell growth [71,72]. However, there is still no result about the prognostic role of FBXW8 in BC. Our results indicated that overexpression of *FBXW8* mRNA was significantly associated with better prognosis in luminal A and basal-like BC patients, however, it was correlated with worse survival in HER2-overexpressiong BC patients.

An accumulation of pathological data have been proved that FBXW7 is a tumor suppressor by targetting various oncogenic proteins, such as Notch, cyclin E, c-Myc, and c-Jun, for degradation [73,74]. Interestingly, FBXW7 expressed in the host microenvironment also suppressed cancer metastasis depending on the FBXW7/NOTCH/CCL2 axis [75]. Hence, FBXW7 mutation, resulting in loss-of-function of FBXW7, was frequently observed amongst primary human cancers. Approximately 6% of human cancers were FBXW7 mutated, and 9% of primary endometrial cancers were FBXW7 mutated [76]. Reduced expression of FBXW7 has been reported to be correlated with worse outcomes in several human cancers, including gastric cancer [77], colorectal cancer [78], cervical squamous carcinoma [79], glioma [80], and prostate cancer [81]. For BCs, FBXW7 was significantly down-regulated, knockdown of FBXW7 in BC cells promoted cell proliferation, migration, and inhibited cell apoptosis [82,83]. Inactivation of FBXW7 by promoter-specific methylation was correlated with poorly differentiated BC [84]. *FBXW7* mRNA expression was reduced in BC patients with high histological grade and hormone receptor-negative tumors [85]. A meta-analysis including 1900 patients indicated that the prognostic value of FBXW7 at mRNA level in BC was depending on ER status and molecular subtypes [86]. According to our results, increased mRNA expression of FBXW7 was associated with better OS only in grade II BC patients.

Amongst the large family members of F-box, only few members have been extensively studied. Here, we used the KM plotter database to assess the prognostic values of the selected 15 members of *F-box* mRNA expression in BC and demonstrated that FBXO1, FBXO31, SKP2, and FBXO5 were significantly associated with worse prognosis in BC patients. FBXO4 and β-TrCP1 were found to be correlated to better OS. These associated results provide new insights into F-box members in the development and progression of BC. Further studies are needed in order to get detailed understanding of functional characterization of each F-box member and determine whether they can be potential treatment targets of BC.

## Supporting information

**supplementary Figure S1:** 

**supplementary Figure S2:** 

**supplementary Figure S3:** 

**supplementary Figure S4:** 

**supplementary Figure S5:** 

## Abbreviations

APC/C: anaphase-promoting complex/cyclosome
β-TrCP1: β-transducin repeats containing proteins
BC: breast cancer
BCL-6: B-cell lymphoma 6 protein
CCL2: chemokine (C-C motif) ligand 2
CI: confidence interval
FBXO3: F-box only protein 3
GEO: gene expression omnibus
HPK1: hematopoietic progenitor kinase1
HR: hazard ratio
KM plotter: Kaplan–Meier plotter
MEF: mouse embryonic fibroblast
OS: overall survival
SCFise: SKP1–cullin1–F-box protein
SKP1: S-phase kinase-associated protein 1
TNBC: triple-negative breast cancer
WNT: wingless/integrated
