# Supplementary figures and images for: Prognostic values of F-box members in breast cancer: an online database analysis and literature review
Source: Biosci Rep. 2019 Jan 3;39(1):BSR20180949. doi: 10.1042/BSR20180949 (PMC6328874; doi:10.1042/BSR20180949)

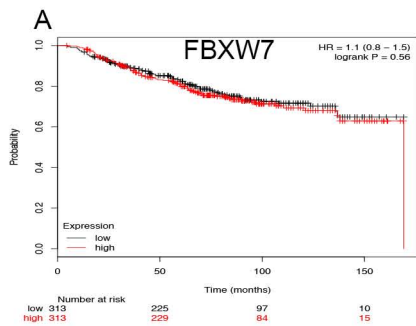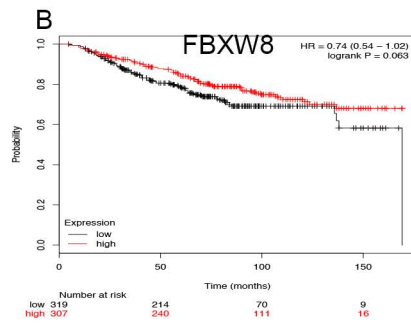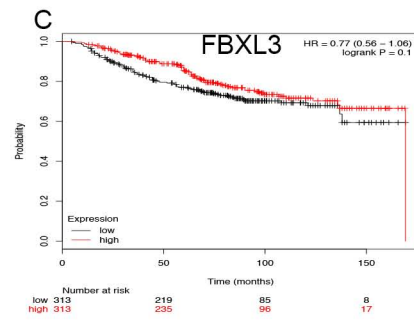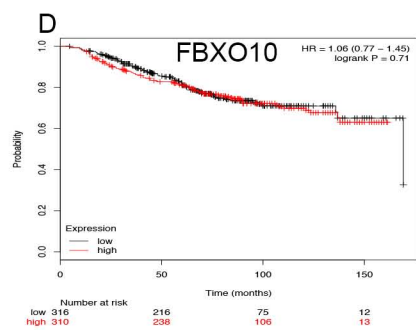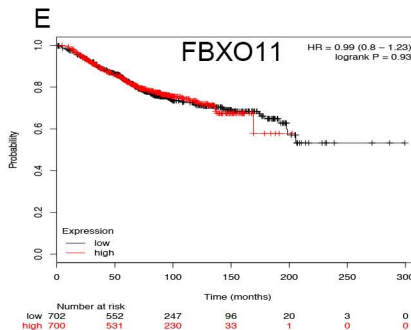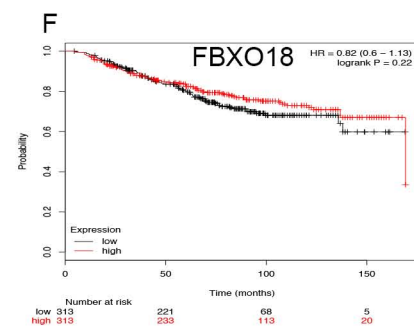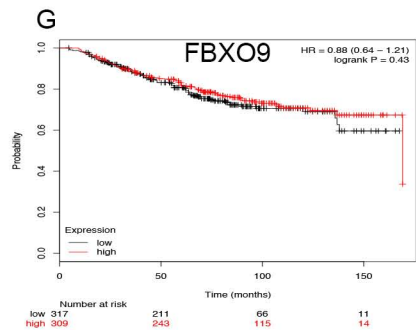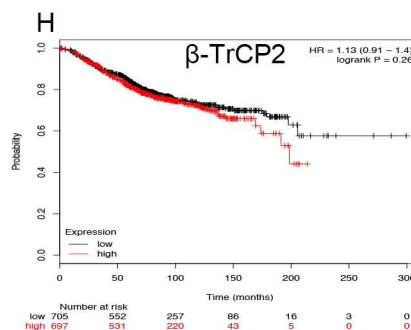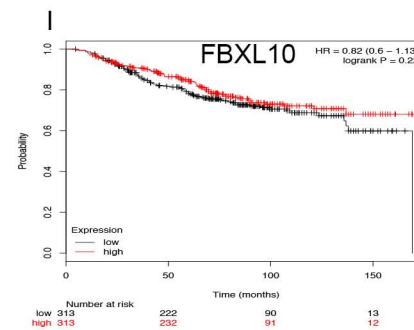

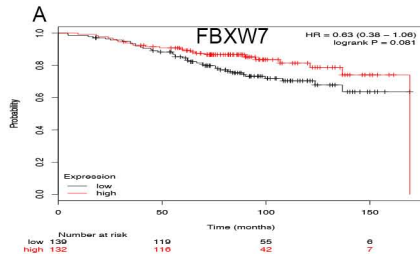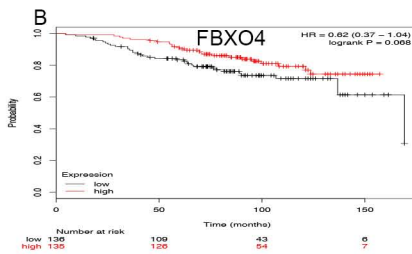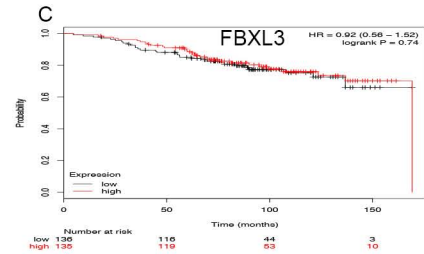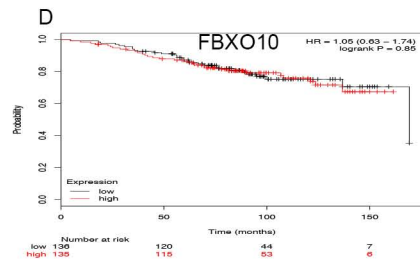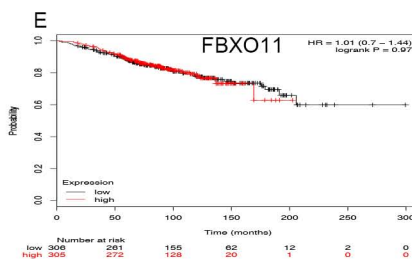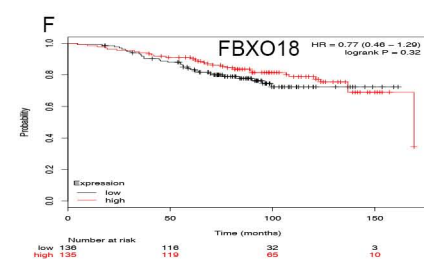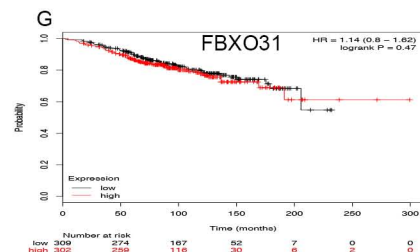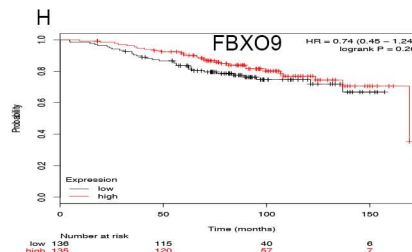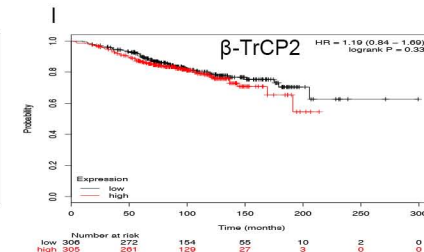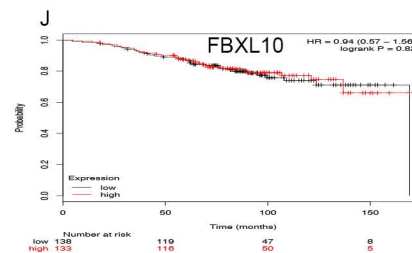

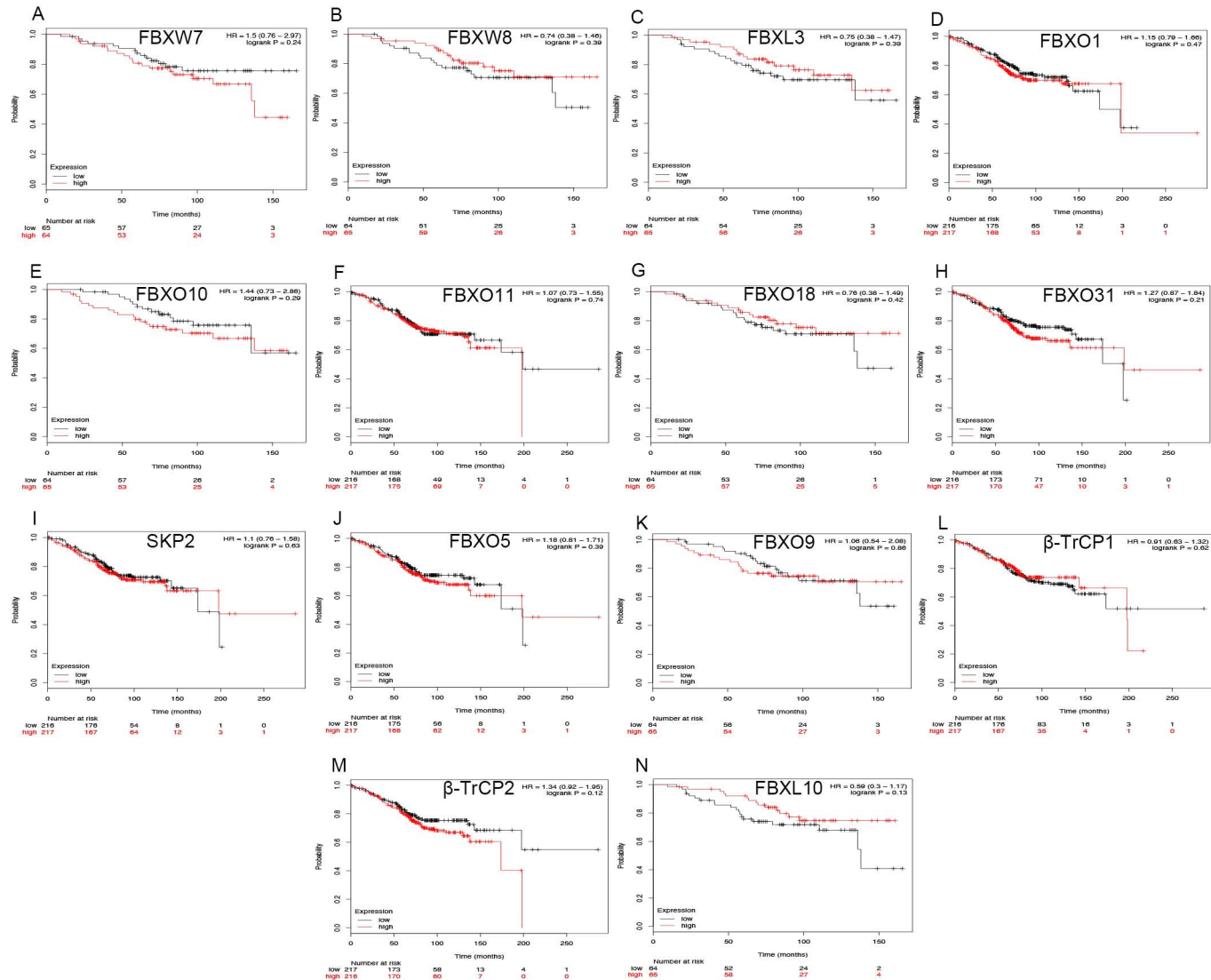

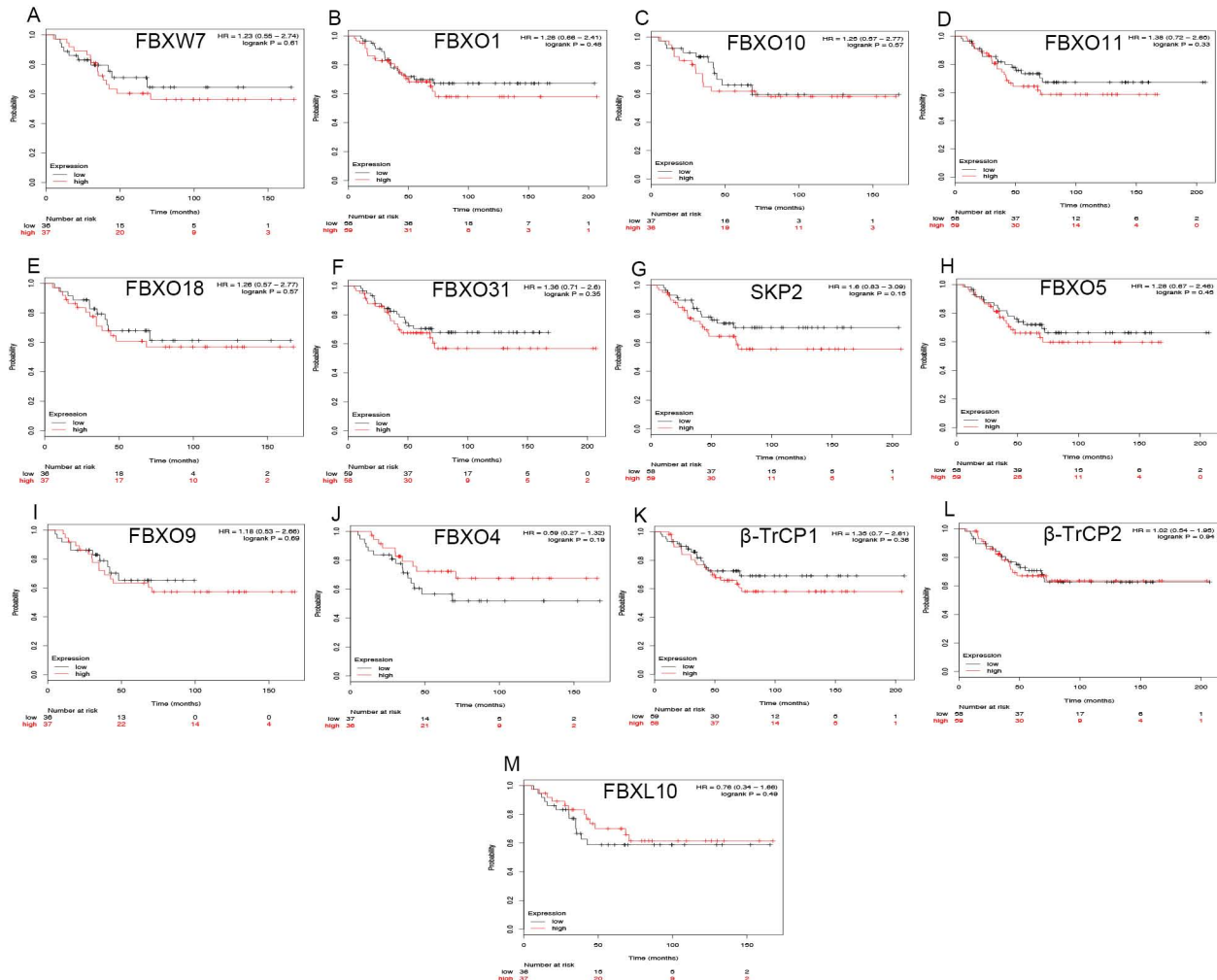

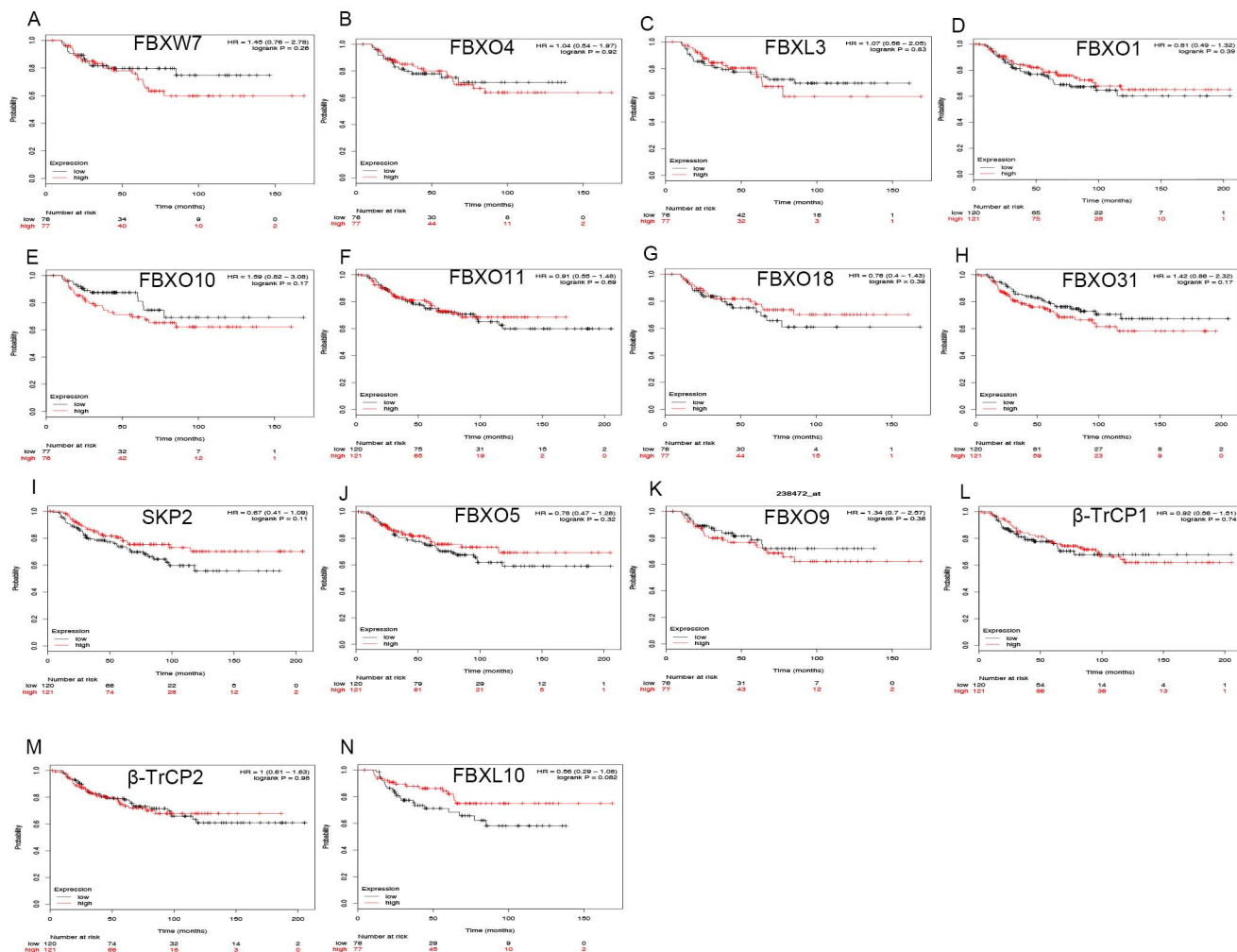

Supplement: Supplementary file 1 [file bsr20180949_Supp1.pdf]
